# Fungal Infections among Psoriatic Patients: Etiologic Agents, Comorbidities, and Vulnerable Population

**DOI:** 10.1155/2021/1174748

**Published:** 2021-09-15

**Authors:** Mostafa Chadeganipour, Shahla Shadzi, Rasoul Mohammadi

**Affiliations:** ^1^Department of Medical Parasitology and Mycology, School of Medicine, Isfahan University of Medical Sciences, Isfahan, Iran; ^2^Infectious Diseases and Tropical Medicine Research Center, Isfahan University of Medical Sciences, Isfahan, Iran

## Abstract

**Background:**

Psoriasis is a chronic inflammatory disorder of the skin and joint, affecting nearly 2-3% of the general population. It is assumed that imbalance between the types of natural microflora can accelerate the onset of the disease. Some fungi can play the role of superantigens and prolong chronic inflammation in the skin of psoriatic patients. The aim of the present investigation was to identify fungal species isolated from patients with psoriasis.

**Methods:**

From March 2016 to May 2019, 289 patients with prior diagnosis of psoriasis were included in this survey. Direct microscopy with potassium hydroxide (KOH 10%), culture, urea hydrolysis, hair perforation test, and growth on rice grains were used to identify clinical isolates, phenotypically. For molecular identification of *Candida* species and *Malassezia* species, PCR-RFLP and PCR-sequencing were used, respectively.

**Results:**

Forty-six out of 289 psoriatic patients had fungal infections (15.9%). Dermatophytes (54.3%), *Candida* spp. (19.5%), *Malassezia* spp. (15.2%), *Aspergillus* spp. (6.5%), and *Fusarium* spp. (4.3%) were the causative agents of fungal infections. Among *Malassezia* and *Candida* species, *M. restricta* (10.8%) and *C. glabrata* (8.7%) were the most prevalent species, respectively.

**Conclusion:**

Our findings suggested that fungal pathogens, particularly dermatophytes, may play an important role in the pathogenicity of psoriasis. Also, due to the high rate of yeast colonization in the clinical samples of psoriatic patients, concomitant use of anti-inflammatory drugs and antifungals may represent an effective therapeutic approach for better management of chronic lesions among these patients. Mycological tests should be applied to indicate the incidence of fungal diseases in psoriatic patients.

## 1. Introduction

Psoriasis is an immune-mediated inflammatory disorder affecting 2-3% of the general population [1]. Dysregulation of the immune system such as keratinocyte hyperproliferation and infiltration of inflammatory cells, especially monocytes, neutrophils, dendritic cells, and T lymphocytes in the epidermis and dermis, is now considered as a decisive phenomenon in the pathogenesis of psoriasis [2]. Although the nature of the microbial antigen or autoantigen that triggers psoriatic T cells remains contentious, interactions among Th1, Th17, Th9, Th22, and Treg cells seem to be important factors for the progression of psoriasis [3]. Also, endotoxins of Gram-negative bacteria, endogenous bacteria, dermatophyte infections, and intestinal yeasts may be involved in the development of psoriasis [4–6]. Psoriasis is a multiorgan dysfunction that commonly occurs in patients with obesity, systemic arterial hypertension, type 2 diabetes, cardiovascular diseases, anxiety, and nonalcoholic fatty liver disease [7, 8]. The objective of the present investigation was to assess fungal infections among psoriatic patients and the identification of etiologic agents by phenotypic and molecular techniques.

## 2. Materials and Methods

This is a cross-sectional study conducted at a mycology reference laboratory (Shefa Lab.) in Isfahan, Iran. Between March 2016 and May 2019, 289 patients with prior diagnosis of psoriasis who revealed nail or skin changes were included in this survey. Psoriasis was diagnosed and confirmed by dermatologists at the specialized clinics. Patients taking topical or systemic antifungal agents in the previous 15 days were excluded from the study (*n* = 17). Age, sex, job, location on the body, and clinical manifestations were documented for each patient. This research was approved by the Ethics Committee of Isfahan University of Medical Science (no. IR.MUI.MED.REC.1398.634), and written informed consent was obtained from all patients.

### 2.1. Conventional Methods

Nail clippings and skin scrapings were collected in sterile Petri dishes for direct microscopic examination (DME) and culture. Potassium hydroxide (KOH) 10% and 20% + dimethyl sulfoxide (DMSO) were used for DME of skin scrapings and nail clippings, respectively. Sabouraud dextrose agar with chloramphenicol and cycloheximide (Mycobiotic agar; Difco, Detroit, MI) (for dermatophyte spp.), Czapek–Dox agar (QUELAB, Quebec, Canada) (for *Aspergillus* spp.), Sabouraud glucose agar (Difco, Detroit, MI), and Dixon's agar (HiMedia, India) (for *Malassezia* spp.) were applied for culture and incubated at 30°C and 37°C. Cultures were examined continuously for the fungal growth up to 4 weeks. Additional diagnostic tests such as urea hydrolysis (QUELAB, Canada), culture on nutritional media (Trichophyton agars; BIOMARK, India), hair perforation test, and growth on rice grains were used to confirm primary identification of dermatophyte spp. [9, 10].

### 2.2. Molecular Identification of *Candida* Species

#### 2.2.1. DNA Extraction

Genomic DNA was extracted using the boiling method [11]. In brief, a loopful of fresh colonies was suspended in 80 *μ*L of double distilled water and boiled for 10 minutes and then centrifuged for 6 minutes at 6500 rpm. The supernatant containing DNA was used for PCR.

#### 2.2.2. Polymerase Chain Reaction-Restriction Fragment Length Polymorphism (PCR-RFLP)

ITS1-5.8SrDNA-ITS2 region was amplified by a PCR mixture containing 5 *μ*L of 10  × reaction buffer, 1.5 mM MgCl_2_, 0.4 mM dNTPs, 30 pmol ITS1 (5′-TCC GTA GGT GAA CCT GCG G-3′) and 30 pmol ITS4 (5′-TCC TCC GCT TAT TGA TAT GC-3′) primers [12], 2.5 U of Taq polymerase, and 3 *μ*L DNA in a final volume of 50 *μ*L. The PCR cycling conditions were as follows: an initial denaturation phase at 95°C for 5 min, followed by 30 cycles of denaturation at 95°C for 30 s, annealing at 55°C for 45 s, and extension at 72°C for 1 min, with a final extension phase at 72°C for 7 min. PCR products were digested with the *Hpa*II (*Msp*I) restriction enzyme (Fermentas, Vilnius, Lithuania). Five microliter of each PCR products and 10 *μ*L of RFLP amplicons were separated by gel electrophoresis on 1.5% and 2% agarose gel (containing 0.5 *μ*g/mL ethidium bromide), respectively.

### 2.3. Molecular Identification of *Malassezia* Species

#### 2.3.1. DNA Extraction

Genomic DNA was extracted from the skin scrapings and dandruff or from colonies subcultured on Dixon's agar (HiMedia, India) by using glass beads and phenol/chloroform techniques [13, 14]. In brief, a loopful of the skin scale or dandruff was transferred to a 1.5 mL Eppendorf tube, including 300 *μ*L glass beads and 300 *μ*L lysis buffer (200 mM Tris/HCl with a pH of 7.5, 25 mM EDTA, 0.5% SDS, and 250 mM NaCl). Afterwards, the specimens were centrifuged for 1 min at 7,000 rpm and then 300 *μ*L of phenol/chloroform was added, followed by vortexing and centrifugation for 6 min at 5,000 rpm. In the following, the supernatant was transferred to a new tube, and the same amount of chloroform was added to it and centrifuged for 7 min at 6,000 rpm. Subsequently, the supernatant was transferred to a new tube, and then alcohol (2.5 times) and 3 M sodium acetate (1/10 volume) were added and stored at −20 °C for 1 h and centrifuged for 5 min at 10,000. The supernatant was removed, and 500 *μ*L alcohol 70% was added to the pellet, which was then centrifuged for 12 min at 12,000 rpm. At the final stage, the supernatant was discarded, and 50 *μ*L double distilled water was added and kept at −20 °C.

#### 2.3.2. Amplification of D1/D2 Region of 26S rDNA

PCR reaction included 5 *μ*L of 10 × PCR buffer, 1.5 mM MgCl_2_, 0.5 mM of each forward (5′-TAACAAGGATTCCCCTAGTA-3′) and reverse (5′-ATTACGCCAGCATCCTAAG-3′) primers [15], 0.2 mM of each deoxynucleoside triphosphate, 1.25 U of Taq polymerase, and 2 *μ*l template DNA in a final volume of 50 *μ*l. The PCR conditions were as follows: an initial denaturation step at 94°C for 5 min, followed by 34 cycles of denaturation at 94°C for 45 sec, annealing at 55°C for 45 sec, and extension at 72°C for 1 min, with a final extension step of 72°C for 7 min. The PCR products were visualized by 1.5% (w/v) agarose gel electrophoresis in TBE buffer, stained with SYBR Safe DNA gel stain (1 : 10,000 dilution in TBE), and photographed under ultraviolet transilluminator (UVITEC, UK).

#### 2.3.3. Sequencing

All amplicons were subjected to sequence analysis. They were purified by the ethanol purification method, and cycle sequencing reactions were performed in a forward direction (Bioneer, South Korea). The sequencing products were evaluated with Chromas 2.4 (https://chromas.software.informer.com/2.4/) and analyzed using the NCBI BLAST searches against fungal sequences existing in DNA databases (https://blast.ncbi.nlm.nih.gov/Blast.cgi).

### 2.4. Statistical Analysis

Chi-square and Fisher's exact tests in the SPSS software version 23 (IBM Corp, Armonk, NY) were applied for analysis. A *p* value less than 0.05 was considered statistically significant.

## 3. Results

**Figure 1:**
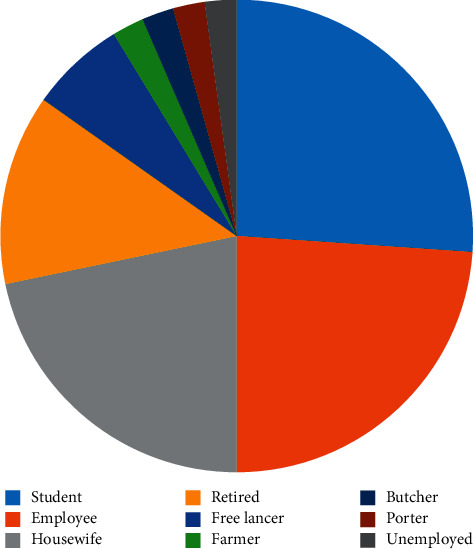
Distribution of psoriatic patients with fungal infections according to their occupation: student (*n* = 12), employee (*n* = 11), housewife (*n* = 10), retired (*n* = 6), freelancer (*n* = 3), farmer (*n* = 1), butcher (*n* = 1), porter (*n* = 1), and unemployed (*n* = 1).

**Figure 2:**
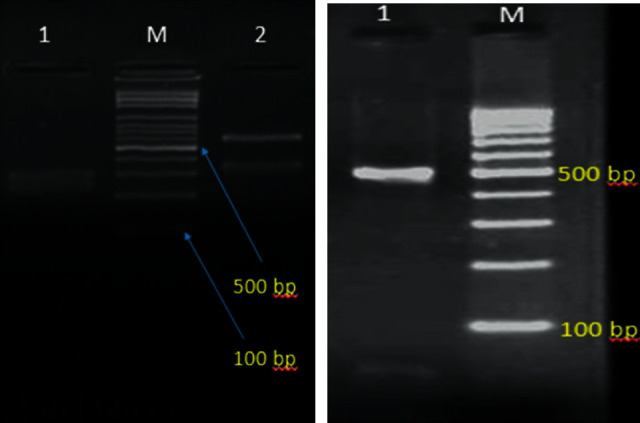
Digested ITS-PCR products with *Msp*I. (a) Lane 1: *C. krusei;* lane M: 100 bp DNA size marker; and lane 2: *C. glabrata*. (b) Lane 1: *C. parapsilosis* and lane M: 100 bp DNA size marker.

Forty-six out of 289 psoriatic patients had fungal infections (15.9%). The male to female ratio of participants was 28/18. The age range of the patients was between 10 and 82 years. The age ranges of 11–20 (23.9%) and 81–90 (2.2%) years had the highest and lowest frequencies, respectively (Table 1). Students were the most commonly infected population (26.1%) followed by employees (23.9%) and housewives (21.7%) (Figure 1). Diabetes mellitus (19.6%), atopic dermatitis (13%), use of corticosteroid (10.9%), and use of wide spectrum antibiotics (8.7%) were the most predisposing factors for fungal infections. Obesity, cardiovascular diseases, and smoking were main comorbid diseases among psoriatic patients (Table 2). Dermatophytes (54.3%), *Candida* spp. (19.5%), *Malassezia* spp. (15.2%), *Aspergillus* spp. (6.5%), and *Fusarium* spp. (4.3%) were the causative agents of fungal infections in the present study (Table 3). Among *Malassezia* and *Candida* species, *M. restricta* (10.8%) and *C. glabrata* (8.7%) were the most prevalent species, respectively. Interestingly, none of the *Candida* species were *albicans* (Figure 2). All sequences of *Malassezia* spp. were deposited the in the GenBank under the accession numbers MT645556, MT645557, MT645569, MT645570, MT645572, MT645573, and MT645587. Fisher's exact test showed that the association between the psoriasis and fungal species was not statistically significant (*p*=0.88).

## 4. Discussion

Over the past decade, the connection between the inflammatory skin disorders and microbiome has been increasingly accepted [16]. It is assumed that imbalance between the types of natural microflora of the skin and mucosa could accelerate the onset of the disease in vulnerable hosts such as patients with autoimmune disorders [17]. Various microorganisms including fungi, viruses, and bacteria can play the role of superantigens (SAgs) that trigger specific T cells and initiate, intensify, and prolong chronic inflammation in skin disorders [18]. For example, it has been proven for *Staphylococcus aureus* skin colonization in psoriasis and atopic dermatitis [19, 20]. Similar to bacteria, many fungi have also been recognized in encouraging skin-associated lymphoid tissue. *Candida* species are important part of the human microflora, commonly colonizing the mucosal membranes of genitourinary and gastroesophageal tracts and skin. They cause infection in patients with impaired immune system [21]. Despite the fact that the significant role of the microorganisms in the pathogenesis of inflammatory skin disorders has been remarkably analyzed, this connection has been overlooked in the case of fungi. Excessive growth of *Candida* species has been found on the skin of patients with inflammatory skin disorders such as psoriasis and atopic dermatitis [22]. *Candida* strain antigens, mainly surface proteins of *C. albicans*, have been proven to have superantigen-like sequel, following the polyclonal T cell activation and uncontrolled release of proinflammatory cytokines [23]. In a meta-analysis performed by Pietrzak et al. [21], all analyzed investigations revealed a higher oral colonization by *Candida* among psoriatic patients. They suggested that psoriasis may be one of the systemic disorders that induces oral candidiasis; nevertheless, we did not detect oral colonization or oropharyngeal candidiasis in psoriatic patients in the present study. Picciani et al. showed that 26% of psoriatic patients had oral candidiasis in comparison with the control group [24]. Antimicrobial peptides (AMPs), which are exceedingly produced in the skin of psoriatic patients, can inhibit *Candida* spp. growth [25]; however, Taheri Sarvtin et al. reported that *Candida* species were isolated from skin samples of 15% of psoriatic patients compared to 4% of healthy individuals [26]. We isolated *Candida* spp. from skin scrapings of 4.3% of patients with psoriasis. *Candida* species, mainly *C. albicans*, are the most prevalent pathogens isolated in clinical samples of patients with psoriasis vulgaris [4], but none of *Candida* species were *albicans* in the present survey (Figure 2). Although males and females are identically affected by psoriasis vulgaris, within the younger patients, females are more likely to be affected than males [2]. Antifungal drugs have been shown to reduce inflammation in psoriasis [4]; however, we did not evaluate the effects of this variable because the use of antifungal drugs was one of the exclusion criteria in our study. The occurrence of onychomycosis in psoriatic patients is controversial, ranging from 10% to 56% [5, 27]. A possible explanation is that the pathological changes of nails in psoriasis, such as hyperkeratosis, pitting, and onycholysis, are intricate to discern clinically from onychomycosis, and precise assessment relies on mycological tests. High prevalence of onychomycosis among psoriatic patients may related to abnormal capillary unit in psoriatic nails that damages the immune defenses generally supplied by the intact hyponychium and the use of immunosuppressive agents among psoriatic patients. On the other hand, faster turnover of nails in psoriatic patients may be noticed as a significant defense mechanism against fungal invasion [28]. One of the limitations of the present investigation was the lack of control group to compare the rate of onychomycosis in psoriatic patients and control population; nevertheless, the majority of fungal infections in our study belonged to the nail infections (52.2%). This is less than the nail involvement reported by Jendoubi et al. [28] and Zargari et al. [29] which were 71.2% and 69.5%, respectively. In line with our findings, nail pitting is the most common nail matrix involvement among psoriatic patients [30]. Some literatures revealed that 85–90% of patients with psoriasis expand nail involvement in their lifetime [31], but 73% of patients had nail psoriasis in the present study. Leibovici et al. [27] showed a higher prevalence of nail infection in males and elderly patients. In agreement, we also found a higher frequency of onychomycosis in males; however, teens were the most infected population in our survey. Some studies confirm our findings [32, 33], and others deny it [34, 35]. Leibovici et al. reported a higher percentage of dermatophytes as the etiologic agents of fungal infection with the most distribution of *Trichophyton rubrum* (35.4%) among clinical specimens [27], whereas *Epidermophyton floccosum* and *T. interdigitale/mentagrophytes* were the most common dermatophyte species in the present investigation (30%). Although, in some papers, the prevalence of dermatophytosis is lower in psoriatic patients [36, 37], in other studies, the prevalence of this infection is higher among psoriatic patients compared to the control group [5, 38].

## 5. Conclusion

The prevalence of fungal infections among psoriatic patients is controversial. Our findings suggested that fungal pathogens, particularly dermatophytes, may play an important role in the pathogenicity of psoriasis. Also, due to the high rate of yeast colonization in clinical samples of psoriatic patients, concomitant use of anti-inflammatory drugs and antifungals may represent an effective therapeutic approach for better management of chronic lesions among these patients. Furthermore, we found that the occurrence of fungal infections in psoriatic patients is not as uncommon as generally believed. Mycological tests should be applied to indicate the incidence of fungal diseases in psoriatic patients.

## Figures and Tables

**Table 1:** The distribution of psoriatic patients with fungal infections in different age categories.

Age group	Male	Female	Total
0–10	1	1	2
11–20	7	4	11
21–30	4	4	8
31–40	5	1	6
41–50	3	4	7
51–60	4	1	5
61–70	1	2	3
71–80	2	1	3
81–90	1	0	1
Total	28	18	46

**Table 2:** Comorbid diseases in psoriatic patients in the present study.

Comorbid diseases	Number
Obesity	14 (30.4%)
Cardiovascular diseases	9 (19.5%)
Smoking	7 (15.2%)
Nonalcoholic fatty liver disease	5 (10.8%)
Celiac disease	2 (4.3%)
Lymphoma	2 (4.3%)
Anxiety	2 (4.3%)
Parkinson's disease	1 (2.2%)
Unknown	4 (8.7%)

**Table 3:** Etiologic agents of fungal infections isolated from psoriatic patients.

Etiologic agent	Methods of identification	Infected area								
		Hand	Face	Scalp	Nail	Foot	Glabrous skin	Groin	Eyebrow	Total
*T. rubrum*	DM + culture + additional tests^a^	1	0	0	1 (FN) 2 (TN)	1	0	0	0	5
*E. floccosum*	DM + culture + additional tests	0	0	0	3 (TN)	3	0	1	0	7
*T. interdigitale/mentagrophytes*	DM + culture + additional tests	0	0	0	1 (FN) 5 (TN)	2	1	0	0	9
*T. verrucosum*	DM + culture + additional tests	0	1	0	0	0	0	0	0	1
*T. violaceum*	DM + culture + additional tests	0	0	1	0	0	0	0	0	1
*M. gypseum*	DM + culture + additional tests	0	0	1	0	0	0	0	0	1
*M. canis*	DM + culture + additional tests	0	1	0	0	0	0	0	0	1
*C. glabrata*	PCR-RFLP	0	0	0	2 (FN) 1 (TN)	0	1	0	0	4
*C. krusei*	PCR-RFLP	0	0	0	1 (FN)	0	1	0	0	2
*C. parapsilosis*	PCR-RFLP	0	0	0	3 (FN)	0	0	0	0	3
*M. restricta*	PCR-sequencing	0	1	2	0	0	0	0	2	5
*M. globosa*	PCR-sequencing	0	0	1	0	0	0	0	1	2
*Aspergillus* sp.	DM + culture	0	0	0	1 (FN) 2 (TN)	0	0	0	0	3
*Fusarium* sp.	DM + culture	0	0	0	1 (FN) 1 (TN)	0	0	0	0	2
Total		1	3	5	24	6	3	1	3	46

FN: finger nail, TN: toe nail, DM: direct microscopy, and PCR-RFLP: polymerase chain reaction-restriction fragment length polymorphism. ^a^Additional tests including urea hydrolysis, hair perforation test, and growth on rice grains were used to confirm primary identification of dermatophyte species.

## Data Availability

The data on which this research is based are available from the corresponding author upon request. In addition, the sequence data used to support the findings of this study have been deposited in the GenBank repository (https://www.ncbi.nlm.nih.gov/genbank/sequenceids/) under the accession numbers MT645556, MT645557, MT645569, MT645570, MT645572, MT645573, and MT645587.
